# A vision of immuno-oncology: the Siena think tank of the Italian network for tumor biotherapy (NIBIT) foundation

**DOI:** 10.1186/s13046-021-02023-4

**Published:** 2021-07-23

**Authors:** Michele Maio, Michael Lahn, Anna Maria Di Giacomo, Alessia Covre, Luana Calabrò, Ramy Ibrahim, Bernard Fox, The Siena Think Tank, Sergio Abrignani, Allavena Paola, Andrea Anichini, Andrea Ardizzoni, Aversa Gregorio, Mohammad Azab, Marc Ballas, Massimo Barberis, Nicholas L. Bayless, Bryan Bell, Andrea Bifarini, Christian Blank, Petter Brodin, Roberto Camerini, Carbone Ennio, Michele Ceccarelli, Colizzi Francesca, John Connolly, Coral Sandra, Robin Cornelissen, Alexander Eggermont, Joseph Eid, David Fajgenbaum, Elisabetta Ferretti, Soldano Ferrone, Francesca Finotello, Keith Flaherty, Fonsatti Ester, Fratta Elisabetta, Catherine Sautès Fridman, Wolf H. Fridman, Patrick Garcia, Pier Federico Gherardini, Asthika Goonewardene, Graham Hacking, Kevin Heller, Tyler W. Hulett, Imperiale Michael, Daniel Jacobson, Martin Janek, Stefanie Joho, Harold Keer, Samir Kleif, Nikesh Kotecha, Mark Kotter, Nevan Krogan, Antonio Lanzavecchia, Franco Locatelli, Pier-Luigi Lollini, Alberto Mantovani, Alessia Melacarne, Giovanni Melillo, Michael Menden, Daniela Minerva, Lorenzo Moretta, Fouad Namouni, Pier Giorgio Natali, Andrea Necchi, Paola Nisticò, Paga Cosimo, Palmieri Giuseppe, Drew Pardoll, Luis Paz-Ares, Kimberly Plessala, Solange Peters, Robert M. Prins, Olivier Provendier, Rino Rappuoli, Maria Rescigno, Dominik Ruettinger, Barbara Seliger, Alessandro Sette, Sigalotti Luca, Marko Spasic, Giampaolo Tortora, Zlatko Trajanoski, Patrizia Tunici, Chiara Vitale, Jon Wigginton, Mahesh Yadav, Haochen Yu

**Affiliations:** 1 Center for Immuno-Oncology, Medical Oncology and Immunotherapy, University Hospital of Siena, Viale Mario Bracci, 16, Siena, Italy; 2 Italian Network for Tumor Bio-Immunotherapy Foundation Onlus, Siena, Italy; 3 iOnctura SA, Avenue Secheron 15, Geneva, Switzerland; 4 Parker Institute for Cancer Immunotherapy, 1 Letterman Drive, San Francisco, 94012 USA; 5 Earle A. Chiles Research Institute at the Robert W. Franz Cancer Center, 4805 NE Glisan St. Suite 2N35, Portland, OR 97213 USA

**Keywords:** Immunotherapy, Novel treatments, PD1, PD-L1, Melanoma, Mesothelioma, Artificial intelligence, Glioblastoma, Corona virus disease 19 (COVID-19)

## Abstract

**Background:**

The yearly Think Tank Meeting of the Italian Network for Tumor Biotherapy (NIBIT) Foundation, brings together in Siena, Tuscany (Italy), experts in immuno-oncology to review the learnings from current immunotherapy treatments, and to propose new pre-clinical and clinical investigations in selected research areas.

**Main:**

While immunotherapies in non-small cell lung cancer and melanoma led to practice changing therapies, the same therapies had only modest benefit for patients with other malignancies, such as mesothelioma and glioblastoma. One way to improve on current immunotherapies is to alter the sequence of each combination agent. Matching the immunotherapy to the host’s immune response may thus improve the activity of the current treatments. A second approach is to combine current immunotherapies with novel agents targeting complementary mechanisms. Identifying the appropriate novel agents may require different approaches than the traditional laboratory-based discovery work. For example, artificial intelligence-based research may help focusing the search for innovative and most promising combination partners.

**Conclusion:**

Novel immunotherapies are needed in cancer patients with resistance to or relapse after current immunotherapeutic drugs. Such new treatments may include targeted agents or monoclonal antibodies to overcome the immune-suppressive tumor microenvironment. The mode of combining the novel treatments, including vaccines, needs to be matched to the patient’s immune status for achieving the maximum benefit. In this scenario, specific attention should be also paid nowadays to the immune intersection between COVID-19 and cancer.

## Background

The annual Think Tank meeting of the Italian Network for Tumor Biotherapy (NIBIT) Foundation is a forum of experts where current and future developments in basic and clinical research of immuno-oncology (IO) therapies are being discussed [1, 2]. In recent years, the IO experts of the Think Tank focused on understanding why certain tumor types responded to immune checkpoint inhibitors (ICI) while others fail to respond to such immune therapies. For example, patients with mesothelioma or glioblastoma have not benefited from either Programmed cell death protein 1 (PD1/CD279)/Programmed cell death ligand 1 (PD-L1/CD274) or cytotoxic T-lymphocyte-associated protein 4 (CTLA-4) monoclonal antibodies (mAbs) treatments at a comparable rate as patients with Non-small cell lung cancer (NSCLC) or cutaneous melanoma. Furthermore, patients who initially respond to ICI may develop resistance to such treatments. Hence, treatment concepts with new agents are needed to prevent or overcome resistance. One important factor to establish new IO therapies is a continued and comprehensive understanding of the underlying pathology. A deeper understanding of the biology of resistance mechanism should also translate to new biomarkers for assessing early success of new treatments. Finally, the applications of Artificial Intelligence (AI) in drug discovery or clinical development may speed up novel IO therapies. This paper focuses on topics which Think Tank faculty selected to advance the efficacy of cancer immunotherapy. Separately, the topic on neoadjuvant immunotherapy is being reported in a different publication [3].

## NSCLC

### Current status in treatment

#### 1st line metastatic/advanced NSCLC

In the first line setting and in patients with > 50% PD-L1 expression in their tumor, monotherapy of pembrolizumab resulted in a superior Overall Survival (OS) than chemotherapy alone [4]. Adding an IO agent to chemotherapy for the first line treatment is beneficial for patients with < 50% of PD-L1 expression in their tumor tissue [5, 6]. The benefit of adding an IO agent to standard chemotherapy is observed in both, non-squamous and squamous NSCLC. For example, patients with non-squamous NSCLC benefited from the addition of pembrolizumab to a platinum-based chemotherapy containing pemetrexed (KEYNOTE 189 study): at 12 months OS was 69.2% with the pembrolizumab-containing combination vs 49.4% for patients receiving chemotherapy alone (hazard ratio [HR] for death, 0.49; 95% CI, 0.38 to 0.64; *P* < 0.001). Data with longer follow-up (median 18.7 months) showed that these benefits in OS and PFS were maintained across all groups. More importantly, the OS remained significantly higher in the pembrolizumab-combination group (54%) although a substantial number of patients in the control arm received subsequently pembrolizumab monotherapy or an alternative IO therapy. Similar findings were observed for patients with squamous NSCLC (KEYNOTE 407 study). Patients where pembrolizumab was added to standard combination of carboplatin and paclitaxel/nab-paclitaxel had a significantly improved OS and progression-free-survival (PFS): median OS was 15.9 months with pembrolizumab-containing combination and 11.3 months for patients receiving chemotherapy alone (HR = 0.64; 95% CI, 0.49 to 0.85; *P* < 0.001) [6]. Based on these data, pembrolizumab in combination with chemotherapy therapy is now approved for patients with < 50% of PD-L1 status. In contrast, for patients with > 50% PD-L1 expression in their tumor, pembrolizumab monotherapy is a possible choice. These findings are not just limited to PD1 mAb and can also be extended to PD-L1 mAbs [7]. For example, adding atezolizumab to standard chemotherapy in NSCLC (e.g., IMpower150, IMpower130 studies) led to higher OS and PFS compared chemotherapy treatment alone [8–10]. In addition to combinations of IO therapy with standard chemotherapies, combination treatments with only IO-containing drugs have been evaluated. In patients with a PD-L1 expression ≥1% (CheckMate 227), those receiving a combination of ipilimumab and nivolumab had a median OS of 17.1 month versus 14.9 months for patients receiving chemotherapy alone (HR, 0.79; 97.72% CI, 0.65–0.96; *P* = .007) [11].

#### 2nd line metastatic NSCLC

For nivolumab, improved OS, PFS and response rates are seen in NSCLC patients with lower thresholds for PD-L1 expression (≥1%, ≥5%, and ≥ 10%) as evident from the CheckMate 017 and CheckMate 057 studies which compared nivolumab monotherapy to docetaxel in the second-line setting [12–14]. Similar observations were reported for pembrolizumab (KEYNOTE 010 study), however with the main difference that patients with ≥50% PD-L1 expression had better OS than patients with ≥1% PD-L1 expression [15].

#### Adjuvant NSCLC

The PD-L1 mAb durvalumab was evaluated in patients with Stage III NSCLC and who were eligible for chemoradiotherapy (PACIFIC trial). In this Phase 3 trial adding durvalumab as a treatment after chemoradiotherapy led to an improved OS and this effect seemed not to be dependent on PD-L1 expression [16]. A subsequent OS analysis reported that patients with ≥1% PD-L1 expression benefited more than those with no or less < 1% of PD-L1 expression [17].

### Considerations for future NSCLC studies

First, to improve future IO therapies sequencing the components of combination therapies may optimize the patient’s immune response. In NSCLC, mechanisms of immune surveillance are present and hence strategies to revitalize cytotoxic T cell responses are needed. While humoral and cellular immune responses against large number of non-mutated shared cancer antigens may be present, the antigen presentation mechanism (= immunoproteasome) is down-regulated [18]. This is particularly observed in NSCLC with mesenchymal phenotype where the restoration of the immunoproteasome by IFN-γ and 5-aza-2′-deoxycytidine improves the immune responses [18]. Based on these concepts, specific treatment steps are needed to first overcome the immune suppression, followed by steps to invigorate the immune response. One such example is perhaps the PACIFIC trial. In this trial chemoradiotherapy-induced cell death presumably led to an immunogenic response [19] which subsequently boosted the immune system for the effect of the PD-L1 mAb durvalumab. Although, the recent study CheckMate 9LA is not a sequential design, it did limit chemotherapy to 2 cycles when combined with nivolumab plus ipilimumab [20]. The addition of 2 cycles chemotherapy to standard IO therapy resulted in a median OS of 15.6 months (95% CI 13.9–20.0) versus 10.9 months (9.5–12.6) in the control group (HR 0.66 [95% CI 0.55–0.80]). Similar to the chemoradiation in the PACIFIC trial, it is possible that the 2 cycles of chemotherapy induced an immunogenic response which aided the ongoing IO therapies. Thus, chemotherapy may function as a booster for IO therapies.

Second, to improve future IO therapies is to select patients appropriately. Today, the immunohistochemistry (IHC) assay for PD-L1 in tumor specimen is the most common assay to determine the choice of therapy in 1st line treatment of NSCLC. The PD-L1 IHC assays were co-developed with their respective PD-1 or PD-L1 mAb and are part of the approved indication [21]. The cut-offs were empirically developed and may not be precise enough for patient selection. For example, in the ATLANTIC study, response outcomes were measured on the basis of < 25% and ≥ 25% PD-L1 expression [22], and ≥ 25% PD-L1 expression was considered a clinically important threshold [23]. By contrast, pembrolizumab monotherapy treatment is determined by a PD-L1 expression of more than 50%. Apart from the differences in assay and clones used for the IHC assay, PD-L1 expression is subject to significant modifications with time in the tumor microenvironment (TME). Hence, changes in the TME may in turn affect the PD-L1 expression. Additionally, PD-L1 expression in the absence of PD-1^+^ effector T cells is unlikely to provide benefits as shown by a retrospective meta-analysis [24]. Prospective studies using validated multiplex ICH assays will clarify the importance of different immune markers, including PD-L1.

Finally, in NSCLC the biopsy material can be limited and thus a proper diagnostic work-up difficult. Based on these observations, patients may still benefit from IO therapies even in the absence PD-L1 tumor expression. Despite these uncertainties, the use of 50% as a cut-off was recently re-affirmed in a 1st line study with the PD1 mAb cemiplimab [25]. While the median OS was not reached (95% CI 17.9-not evaluable), the patients treated with chemotherapy alone had a median OS of 14.2 months (11.2–17.5; hazard ratio [HR] 0.57 [0.42–0.77]; *p* = 0.0002).

Third, to improve future IO therapies the duration of immunotherapy must be optimized. Study CheckMate 153 evaluated patients with previously treated advanced NSCLC and who were receiving nivolumab either as a continuous or a fixed-duration treatment of 1 year [26]. Patients treated continuously with nivolumab had an improved PFS compared to those with interrupted treatments (HR = 0.42, 95% CI 0.25–0.71). On the other hand, long-term follow-up from the KEYNOTE-010 study (*N* = 1033 patients) indicate that discontinuation of pembrolizumab after 2 years did not adversely affect OS. While preliminary, patients who received for 2 years pembrolizumab had an OS rate of 99% OS at 3-years [27]. Alternatively, switching IO agents for the re-challenge (e.g., starting pembrolizumab following previous nivolumab therapy) may be useful [28]. Think Tank members remarked that there is also a lack of biomarker information which could otherwise help in the decision making. For example, markers associated with complete response (CR)/ partial response (PR) and patient’s immune status may provide such an objective decision-making point to stop IO therapy.

Fourth, combinations of novel or current IO therapies may also improve current treatment options. Surprisingly, the combination of ipilimumab and pembrolizumab in patient with more than 50% PD-L1 expression did not result in an improvement in OS [29]. The lack of detecting a benefit may perhaps be more due to the toxicity of combining pembrolizumab and ipilimumab and thus limit the potential of delivering an improved benefit to patients. Hence, combination with reduced toxicity profile may have a greater importance in the future. For example, the TIGT mAb tiragolumab combined with atezolizumab did not significantly add to the known toxicity of atezolizumab: treatment-related adverse events were 80.6% in the combination of tiragolumab/atezolizumab compared to 72% in the atezolizumab monotherapy. At the same time, ORR and median PFS of 37.3% and 5.6 months in the tiragolumab/atezolizumab arm versus 20.6% and 3.9 months in the control arm with atezolizumab alone promise to increase anti-tumor responses in NSCLC [30] Table 1. The ongoing Phase 3 SKYSCRAPER-01 study is expected to have primary endpoint data in mid 2022 (NCT04294810).

**Table 1:** Select Immuno-Oncology Therapies Discussed at NIBIT Think Tank Meetings 2018–2020

Key Therapies up to 2019	Ref	Key Recent Developments 2019/2020	Ref
**NSCLC**			
Pembrolizumab for > 50% PDL1	[4]	Cemiplimab for > 50% PDL1	[25]
Atezolizumab for > 50% PDL1	[7]		
Pembrolizumab +chemotherapy for < 50% PDL1	[5, 6]	Nivolumab/Ipilimumab + 2 cycles chemotherapy	[20]
		Atezolizumab + tiragolumab	[30]
		Pembrolizumab + ipilimumab (No Improvement in > 50% PD-L1^+^ Patients)	[29]
**Cutaneous Melanoma**			
• BRAF/MEK • Nivolumab/Ipilimumab • Pembrolizumab	[31]	Pembrolizumab + lenvatinib	[32]
Ipilimumab +Fotemustine in brain metastasis	[33]	Atezolizumab was added after 1 cycle of vemurafenib and cobimetinib	[34]
Ipilimumab + guadecitabine	[35]		
**Mesothelioma**			
Pemetrexed/Platinum	[36]	Tremelimumab + durvalumab	[37–45]
		Nivolumab + ipilimumab	[46]
		Nivolumab	[47]

## Melanoma

### Current status in treatment

#### Metastatic/advanced melanoma – 1st line treatment

Checkpoint inhibitors targeting CTLA-4 and PD-1 are recommended in patients who have no BRAF mutation, while patients with BRAF mutation should first be treated with BRAF/MEK inhibitors [31]. In general, the decision to use IO therapies in melanoma is less dependent on the PD-L1 expression level than in NSCLC. Ipilimumab alone is an effective treatment in melanoma [48] but when compared to the efficacy and toxicity profile of PD1 inhibitors [49, 50], there is a preference to use pembrolizumab or nivolumab as first line therapy. Today, for the combination therapies lower doses of ipilimumab are recommended [51]. Combination therapy with nivolumab and ipilimumab may also be an option for patients with untreated brain metastases [52–55]. In this context, initial data from the (NIBIT)-M1 phase II study indicated that ipilimumab in combination with chemotherapy fotemustine showed efficacy in advanced melanoma, including patients with brain metastases [33, 56], opening the path to subsequent studies in this specific subset of melanoma patients [57].

Furthermore, meta-analysis of the CheckMate studies showed that patients with CR had a greater OS than patients with PR or stable disease (SD). For example, 3-year OS was 94% for patients achieving a CR compared to 45% for those achieving a PR [58]. For patients with BRAF^V600E/K^ mutant melanoma the first line treatment is generally a BRAF/MEK inhibitor, and upon progression patients may receive IO therapy as 2nd line treatment. Initiating the therapy with a BRAF/MEK inhibitor is expected to deliver a rapid anti-tumor response compared to an IO-therapy. However, the duration of responses may be shorter with a BRAF/MEK inhibitor alone compared to ICI [59].

#### Metastatic/advanced melanoma – 2nd line treatment

Patients who had prior BRAF/MEK inhibitors generally receive monotherapy with PD1 inhibitors and in select patients a combination of nivolumab and ipilimumab [60, 61]. For patients without a BRAF^V600E/K^ mutant melanoma the initial therapy may be either nivolumab or pembrolizumab monotherapy. On progression, either another PD1-inhibitor may be given or a combination of nivolumab and ipilimumab. After these therapies are exhausted, clinical trials are currently the only option for nearly 50% of all melanoma patients.

### Considerations for future studies in patients with cutaneous melanoma

One potential improvement of current IO therapies may be obtained by sequencing current therapies rather than given all combination agents concomitantly. For example, patients with BRAF^V600E/K^ mutant melanoma may benefit from a short initial treatment with a BRAF/MEK inhibitor (e.g., 2 weeks) followed by checkpoint inhibitors. This approach may reduce the risk of toxicity associated with long-term use of BRAF/MEK inhibitors and also sensitize the immune system to subsequent IO therapies [34, 62, 63]. The potential benefit of such a triplet treatment was also observed for the PD-L1 inhibitor atezolizumab with BRAF/MEK inhibitors [34]. In this Phase 3 study, patients received vemurafenib and cobimetinib for the first cycle before they were randomized to atezolizumab or placebo. The experimental arm reported a PFS of 15.1 months for the triplet versus 10.6 months for the control am (hazard ratio = 0.78; 95% CI 0.63–0.97; *p* = 0.025) [34].

To consider intermittent or alternating combination therapies with novel agents, it is important to first establish whether shorter ICI therapies can be given without a negative impact on clinical outcome. Long-term follow-up data from the KEYNOTE 001 and 006 studies [64, 65] suggest that patients with CR and PR may safely discontinue treatment after 2 years. Shorter treatment schedules with ICI are not associated with a risk of progression [66]. However, there is a higher risk of progression in those patients who fail to achieve a CR [67]. To further validate this concept, studies are currently evaluating the benefit/risk of early discontinuation (e.g., the STOP-GAP study) [68]. Upon relapse, retreatment is available albeit at the risk of being less effective [67]. In such situation, the combination therapy of nivolumab and ipilimumab may be the appropriate option for re-treatment.

Novel combinations in patients who relapse or are refractory to current therapies are needed. For example, the combination of pembrolizumab and the kinase inhibitor lenvatinib showed an ORR of 21.4% (95% CI 13.9–30.5; 2 CRs, 20 PRs) and 31.0% (15.3–50.8; 1 CR, 8 PRs) for patients with PD on prior ICI. Median (95% CI) PFS and OS were 4.2 months (3.5–6.3) and 13.9 months (95% CI 10.8-NR), respectively [32]. A similar study combining atezolizumab with bevacizumab in melanoma patients is currently ongoing and results may be expected in mid 2021 (NCT04091217). This approach of combining anti-angiogenic compounds with ICI seems to be supported by a meta-analysis of past combination studies [69] Table 1.

## Mesothelioma

### Current status in treatment of mesothelioma

#### Advanced/metastatic mesothelioma - 1st and 2nd line studies

Mesothelioma is characterized by an immune-suppressive TME, which may contribute to resistance of current therapies [70]. Initial monotherapy studies with the anti-CTLA-4 agent tremelimumab opened the field of ICI therapy in second line mesothelioma patients [37–41]. However, the phase III DETERMINE study with single agent tremelimumab showed no survival benefit over placebo in this clinical setting [42]. Instead, monotherapy with nivolumab had an ORR of 24% [43] and pembrolizumab given as a 2nd line treatment had a response rate of 20% in the KEYNOTE 028 study [44]. This latter trial led to the phase 3 PROMISE-Meso study that showed a median OS of 10.7 months for pembrolizumab vs 11.7 months for gemcitabine or vinorelbine chemotherapy (*p* = 0.85) [45]. The investigators, however, suspected that patients on the control arm may have switched to IO therapies once they progressed, which subsequently confounded the survival endpoint [45]. Additionally, in the 2nd line therapy nivolumab had a longer OS with a median of 9.2 versus 6.6 months on placebo (HR = 0.72; 95% CI, 0.55–0.94; *P* = 0.02) and investigator-assessed PFS was longer for nivolumab versus placebo (median PFS of 3.0 versus 1.8 months; HR 0.62; 95% CI, 0.49–0.78; *P* < 0.001) [47].

Switching from monotherapy to CTLA-4 and PD-1/−L1 combinations, the NIBIT-MESO-1 study demonstrated an ORR of 28%, a DCR of 65%, a median OS of 16.5 months [71] and paved the way to combination regimens targeting CTLA-4 and PD-1/−L1. The combination of nivolumab and ipilimumab (INITIATE study) showed PR in 29% (10/34) and SD in 38% (13/34) as 2nd line treatment [72]. Most recently, the phase III study CheckMate 763 with the combination of nivolumab and ipilimumab in 1st line therapy demonstrated a median OS of 18.1 months (95% CI: 16.8–21.4) versus 14.1 months (12.4–16.2; hazard ratio 0.74, 96.6% CI 0.60–0.9; *p* = 0.0020) for patients receiving conventional chemotherapy [46]. These results led to the approval of the combination nivolumab and ipilimumab by the FDA as first line therapy of mesothelioma patients after almost three decades of chemotherapy being the standard of care [36] Table 1.

### Considerations in future mesothelioma studies

Because mesothelioma has an aggressive tumor growth, combination therapies are the most likely path to improving the treatment of mesothelioma. Targeting the underlying immune-suppressive TME with novel agents, specifically by altering the biology of Myeloid Derived Suppressor Cells (MDSCs), was felt to be a key next step. However, MDSCs-directed agents such as the PI3k γ/δ inhibitor eganelisib (IPI-549) have not produced convincing clinical benefit [73]. Additionally, the potential role of the Tumor Mutational Burden (TMB) and of ICI therapy rechallenge needs to be further explored in mesothelioma, as most recently demonstrated by the results of the long-term follow-up of patients enrolled in the NIBIT-MESO-1 study [39].

## Glioblastoma Multiforme (GBM)

### Current status in treatment

GBM patients harbour an immuno-suppressive TME and several studies with ICI have been conducted to overcome this immuno-suppressive TME [74]. PDL-1 expression is present in at least half of GBM patients [75]. In the past three decades, standard therapy in the 1st line treatment of GBM includes surgery followed by radio-chemotherapy with temozolomide, where median OS is approximately 16 months or shorter, depending on the areas of resection [76], while 2nd line treatment includes regorafenib, lomustine, bevacizumab and/or radiotherapy [77]. To date, phase 3 studies with ICI therapy have not demonstrated OS benefits in GBM patients enrolled in the CheckMate 143 study [78, 79]. Moreover, nivolumab alone or combined with temozolomide and radiation therapy failed to result in a statistically significant improvement in OS in patients with newly diagnosed GBM with or without MGMT promoter methylation in the CheckMate-548 (NCT02667587) and CheckMate-498 (NCT02617589) trials (unpublished).

### Considerations in future GBM studies

Gene expression studies have been used to better understand the pre-existing immune state to potentially recognize a possible mechanism of resistance [80]. For instance, drivers of resistance may include the presence of M2 macrophages, low immunogenic neoantigens and dysfunctional infiltrating T lymphocytes [81]. Enriched condition of PTEN mutations may be additional influence factors for determining response to ICI therapies in GBM [82].

### Immuno-oncology approaches beyond just checkpoint inhibition - epigenetic immunomodulation

Epigenetic modifiers, including hypomethylating agents, show broad immune-modulatory activity in tumor cells and have potential to improve outcomes of cancer immunotherapies [83]. DNA hypomethylating agents include azacytidine, decitabine and guadecitabine (SGI-110) [84]. Azacytidine, decitabine and guadecitabine all induce robust cellular changes capable of enhancing host responses; however, among the three agents azacytidine was shown to be less effective [85]. As such, guadecitabine may enhance established immunotherapy as investigated in the NIBIT-M4 trial [35]. NIBIT-M4 is a phase 1b study (NCT02608437) evaluating guadecitabine in combination with ipilimumab in patients with treatment-naïve unresectable Stage III/IV melanoma (*n* = 19). The most common treatment-related adverse events of any grade were myelotoxicity (Grade 3/4: 79%) and immune-related adverse events (63%). Immune-related disease control was observed in 8/19 patients (42%) with an objective response demonstrated in 5/19 patients (26%) [35]. Median tumor CpG sites methylation was significantly lower at Week 4 and Week 12 compared to baseline. Transcriptome analysis showed higher numbers of differentially expressed genes at Week 4 and Week 12. Commonly activated immunomodulatory pathways included Th1 and Th2 cells, ICOS and CD28 signaling, and IFN-γ pathway. HLA-class I expression was upregulated on tumor cells, while an increase in CD8^+^, PD-1^+^ T cells and CD20^+^ B cells was observed in post-treatment tumor core specimens [35]. Based on these data, a randomized Phase 2 study (NIBIT-ML1) was initiated, where NSCLC and melanoma patients resistant to PD1/PD-L1 therapies will receive either a triplet of nivolumab/ipilimumab/guadecitabine or the doublet of nivolumab/ipilimumab (NCT04250246). The objective is to determine whether the addition of guadecitabine to ICI will reverse the treatment resistance in melanoma and NSCLC patients [86].

### Immuno-oncology approaches beyond just checkpoint inhibition - Cancer vaccines and oncolytic viruses

There are two approved examples of an oncolytic virus and a cancer vaccine: talimogene laherparepevec and sipuleucel-T, respectively. Sipuleucel-T consists of *autologous peripheral-blood mononuclear cells activated with prostatic acid phosphatase and granulocyte-macrophage colony-stimulating factor*. While the personalized vaccine sipuleucel-T is no longer available, because the original manufacturer withdrew the product from the market, companies are interested in revitalizing sipuleucel-T. Other vaccines in melanoma, which are not personalized, include gp100, which was approved when given together with adjuvant montanide and interleukin (IL) – 2 [87].

Talimogene laherparepvec, an intralesional, oncolytic viral therapy using genetically modified herpes virus is approved for the treatment of adults with unresectable melanoma that is regionally or distantly metastatic (stage IIIB, IIIC and IV M1a) with no bone, brain, lung or other visceral disease [88–90]. Its mode of action, including neoantigen release and stimulation of local tumor responses is complementary to IO therapy [91]. Responses are not limited to treated tumor lesions: “off-target” regression of distant metastases were also reported [88]. In addition to talimogene laherparepvec monotherapy, trials in combination with PD1 inhibitors are being conducted [89, 90].

The gp100 vaccine was evaluated by comparing it to ipilimumab alone or in combination with ipilimumab in patients with melanoma [92]. In this study, patients treated with the ipilimumab plus gp100 combination had a median OS of 10.0 months compared to an OS of 10.1 months for patients receiving ipilimumab monotherapy [92]. While both ipilimumab treatment groups had a better OS than patients receiving only the gp100 vaccine, there was a small difference in long-term OS: patients who received gp100 vaccine plus ipilimumab had a less favorable long-term OS than patients receiving ipilimumab alone. This raised a concern that perhaps adding vaccines to IO therapy may have a negative outcome [93]. Preclinical studies suggest that the approach of using montanide as a depot for a peptide antigen vaccine results in T cell sequestration, dysfunction and deletion, providing a possible explanation for the reduced efficacy of the gp100 ipilimumab combination [94]. Hence, further studies are needed to determine the risk of using the adjuvant montanide in vaccine studies.

In patients who respond to immune checkpoint blockade, tumor cells lose their initial neoantigens, while clonal T cell populations expand in proportion to the number of neoantigens lost [95]. As this loss of neoantigens and the T cell clone expansion occurs, the TME changes as determined by gene expression studies. A limitation of this study is absence of information on expression of non-mutated shared antigens, which can also be downregulated in response to immunotherapy with T cells targeting a given antigen [96]. The gene expression of the TME changes from a stromal/tumor-associated to host immunity-associated genes. This implies that patients who respond to ICI may have a pre-existing immune competence that is likely suppressed. Nowhere is this better characterized than in melanoma where the majority of patients have T cell responses to shared melanoma antigens that can be detected in tumor-infiltrating lymphocytes (TIL) following activation and expansion in IL-2. Presence of gp100 reactive TIL in the adoptive immunotherapy product provides a significant improvement in response rate [97]. Consequently, vaccines are thought to enhance or to elicit this pre-existing immune competency.

Another key factor to increase cancer vaccine efficacy is the induction of a global immune response. In the past, vaccine development was narrowly focused on how to best activate T cells. However, vaccines that induce a one-time increase of T and B cells with no sustained antibody and T cell responses, may not be effective [98]. It is well understood that co-stimulatory signals can augment effector and memory immune responses and vaccine combinations with agonists for OX40, GITR and 4-1BB have demonstrated significantly improved responses and apparent cures in animal models [99, 100]. These observations were the basis of initiating clinical trials to determine the usefulness of this approach in patients (NCT02737475, NCT04470024, NCT02451982).

To achieve such a global immune response viral vectors may deliver antigens and amplify the immune responses. Talimogene laherparepvec activates the immune response by releasing tumor-associated antigens and subsequently stimulates the local immune responses. After intra-tumoral injection of talimogene laherparepvec, patients that benefited from the treatment had an increase in CD8^+^ T and NK cells in their original tumor site [101]. One should also be reminded that intra-tumoral injection of other conventional vaccines e.g., influenza vaccine can also induce tumor regression [102]. Therefore, it is possible that viral vectors are broad activators of the immune responses in cancer and thus ideal delivery agents of vaccines.

In addition to viral therapy approaches, cellular therapies are increasingly being studied in patients with solid cancers. One such example is the use of virus-specific T cells (VST), a subset of T cells isolated through a selective activation and expansion process. This approach was used to treat patients with nasopharyngeal cancer, by selecting T cells that are Epstein-Barr virus-specific [103]. In the ongoing Phase 2 studies, the infusion of such VSTs was associated with longer than expected survival [104]. The VSTs are characterized by high number of gene transcripts associated with anti-cytotoxic activity, durability and memory phenotype.

### Immuno-oncology approaches beyond just checkpoint inhibition - role for “big data” and artificial intelligence (AI)

In the past years artificial intelligence (AI) emerged as a new tool for cancer research. The use of AI can be categorized in the following areas: (a) support basic research and target discovery; (b) improve clinical trial designs and outcome; (c) support registration efforts; (d) monitor for safety signals.

There is a growing number of databases (such as The Cancer Genome Atlas, TCGA, Therapeutically Applicable Research to Generate Effective Treatments, TARGET, Cancer Cell Line Encyclopedia, CCLE) which collect data of various tumor types, such as gene expression, genetic mutations and key clinical outcome data. While these databases are often useful to determine whether a specific target is present in human tumors, there are some limitations: (a) tumors evolve and change their genetic and expression profile; (b) oncogenic signaling is often context dependent. Recognizing the limitations of data from large databases collected at a single time point, it is important to create systems where one can mimic conditions that are an approximation of the patient’s dynamic malignant disease.

AI can also help in interpreting existing and designing future clinical studies, especially if original data are based on small patient numbers. In such cases, historic or external databases can be used to supplement the data from such small clinical trials. One such example is the MDACC 2013–0715 trial in patients with renal cell carcinoma (NCT02210117). In this 3-arm study, three different treatments were given in a neoadjuvant setting: nivolumab as monotherapy or in combination with bevacizumab or ipilimumab. Using gene expression data and comparing the information with other databases, an IFN-γ signature was identified which was associated with treatment benefit. Using further bio-informatics approaches, a JAK2 gene expression profile was also identified as a driver for response to PD1 therapy.

Another example is the assessment of various immune subsets using cytometry by time of flight (CyTOF) techniques. Patients with Wilms tumor (WT) were treated with combination chemotherapy (vincristine/actinomycin D) and immune cells (CD45^+^) were evaluated both before and after chemotherapy [105]. After chemotherapy tumor infiltrating NK cells were observed in tumor specimens. Additionally, tumor-infiltrating CD4 and CD8 T cells had increased expression of PD1, HLA-DR and CD57 [105]. In another study, the TCGA database was used to deconvolute gene expression data and to identify immune cell subsets in a range of cancer malignancies [106]. This approach may help to determine which type of tumors harbor immune suppressive conditions.

Based on its increasing application in cancer, AI was recently used to determine whether available drugs may be repurposed as effective treatments for COVID-19. The COVID-19 Registry of Off-label & New Agents project was created to advance such effective treatments, develop optimal clinical trial study design (sample size, target subpopulations), and determine if a drug should move forward to wider clinical use [107]. Furthermore, AI can be used to integrate omics data and to identify new biologic pathway(s) activated in COVID-19-affected patients compared to healthy donors. For example, transcriptomic and proteomic data from different databases, indicate little to no expression in lung tissues of ACE2, the entry receptor conventionally identified for SARS-CoV-2. Thus, these findings imply that SARS-CoV-2 is unlikely to enter host cells via lung tissues. Instead, the initial infection is thought to occur in nasal, oral, and gastrointestinal tissues given their positivity of ACE2 expression. Following this initial step, a secondary infection may occur in the lung due to viral migration through the lymphatic system and bloodstream [108]. Moreover, by combining protein modeling and molecular dynamics simulations, non-conservative substitutions in functional regions of the spike glycoprotein of SARS-CoV-2 were demonstrated to contribute to differences in its virulence [109]. In spite of being potentially useful in the present “pandemic contingency”, AI will undoubtedly prove useful in the future to design novel clinical trials with more inclusive molecular eligibility criteria in cancer patients [110].

## COVID-19 and Cancer

The current SARS-CoV-2 pandemic revealed that subjects with risk factors, such as advanced age, male gender, smoking status, comorbidities of hypertension, diabetes, cardiovascular disease, chronic respiratory disease, are at increased risk of developing COVID-19 and its severe clinical outcomes. Cancer patients with similar risk factors are also at comparable risk in developing COVID-19 as subjects without cancer, in part because of the systemic immunodepression due to anticancer treatments [111, 112]. Results from the COVID-19 and cancer Consortium (CCC19) demonstrated that the 30-day all-cause mortality was associated with general risk factors (such as male sex, increased age) as well as risk factors unique to patients with cancer (such as ECOG performance status, progressive disease) [113]. This was further supported by the UK Coronavirus Monitoring Project Team study, which reported a 30-day mortality rate of 28.5% vs 40.5% for patients with or without cancer, respectively [114]. The type of anti-cancer therapy, however, does not seem to significantly influence mortality except for patients receiving chemo-immunotherapy [114]. Noteworthy, the potential immune intersection between COVID-19 disease and cancer therapy raises important practical clinical questions and highlights multiple scientific gaps yet to be filled [115, 116]. Nevertheless, preliminary evidence suggests that ICI therapy does not worsen the course of COVID-19 disease allowing to continue treating patients [117].

### Future directions

A better understanding of the interaction between COVID-19 and cancer patients is essential to guide their comprehensive care during the pandemic. Interestingly, differential effects of SARS-CoV-2 on different cellular immune components, including a reduction in cytotoxic CD8^+^T cells, NKT cells, effector memory CD4^+^ and CD8^+^ T cells in severe COVID-19 patients, an expansion on NK cells and memory B cells in patients with mild symptoms, and the role of a pre-existing immunity on the severity of COVID-19 disease have been reported [118]. This evidence will need to be swiftly translated to cancer patients not only to gain a better understanding of SARS-CoV-2 infection in cancer but also to investigate the efficacy of COVID-19 vaccination in cancer patients within and outside clinical trials [119].

## Conclusions

The complexity of immune responses to malignancies continues to be an expanding area of basic and clinical research that is additionally challenged by the SARS-CoV-2 pandemic. Multi-omics approaches are becoming a key contributor in drug development [120, 121]. Especially, as publicly available databases on gene expression (transcriptomics), genetic aberrations (genomics), protein and metabolic changes (proteomics and metabolomics) and other cancer-related studies are expanding, AI-based approaches will reveal new treatment concepts for laboratory and clinical investigation [122]. Nowhere is this concept of leveraging multi-omics data more visible as in lung cancer [123]. But, to test such in silico developed hypothesis new laboratory testing formats are needed. For example, organoid or 3D cultures can be used more efficiently to study drug-cell or cell-cell interaction [124]. Such information may help to narrow the choices of potentially effective combination therapies in the clinic. In addition to the choices or the number of drugs, it is also important to identify the best way of administering each component of the combination. Combinations can work as synthetic lethal drug regimen, sequential treatments, alternative or intermittent drug regimens and as multi- or low dose therapies [125]. In these diverse scenarios, the future Think Tank meetings will continue to focus on innovative ways to study drug combinations for patients who are not benefiting from today’s IO therapies.

## Data Availability

Not applicable.

## Abbreviations

AI: Artificial Intelligence
BRAF: v-raf murine sarcoma viral oncogene homolog B1
CAR-T: Chimeric antigen receptor T cells
CCLE: Cancer Cell Line Encyclopedia
CD: Cluster of Differentiation
CI: Confidence Interval
COVID19: Corona Virus Disease 19
CR: Complete Response
CTLA-4: Cytotoxic T-lymphocyte-associated protein 4
CyTOF: Cytometry by time of flight
DNA: Deoxyribonucleic acid
GBM: Glioblastoma multiforme
HLA-class I: Human leukocyte antigen-class I
ICI: Immune checkpoint inhibitors
ICOS: Inducible costimulator
IFN-γ: Interferon-gamma
IL: Interleukin
IO: Immuno-oncology
mAb: monoclonal Antibodies
MEK: mitogen-activated protein/extracellular signal-regulated kinase kinase
MDSCs: Myeloid Derived Suppressor Cells
NIBIT: Network Italiano per la Bioterapia dei Tumori, also known as “Italian Network for Tumor Biotherapy”
NK: Natural Killer cells
NSCLC: Non-small cell lung cancer
ORR: Overall Responses Rate
OS: Overall Survival
PD1: Programmed cell death protein 1
PD-L1: Programmed cell death ligand 1
PFS: Progression Free Survival
PI3K: *Phosphatidylinositol 3-kinase*
PR: Partial Response
PTEN: Phosphatase and tensin homolog
RECIST: Response evaluation criteria in solid tumors
SARS-Cov 2: Severe acute respiratory syndrome coronavirus type 2
SD: Stable Disease
TARGET: Therapeutically Applicable Research to Generate Effective Treatments
TCGA: The Cancer Genome Atlas
Th1/Th2: T helper cells 1 and 2
TIGIT: T-cell Immunoglobulin and ITIM Domain (TIGIT)
TIL: tumor infiltrating lymphocytes
TMB: Tumor mutational burden
TME: Tumor Microenvironment
VST: virus-specific T cells
WT: Wilms Tumor
